# Mixed reality simulation of rasping procedure in artificial cervical disc replacement (ACDR) surgery

**DOI:** 10.1186/1471-2105-11-S6-S11

**Published:** 2010-10-07

**Authors:** Tansel Halic, Sinan Kockara, Coskun Bayrak, Richard Rowe

**Affiliations:** 1 Mechanical, Aerospace and Nuclear Engineering Department, Rensselaer Polytechnic Institute, Troy, New York, USA; 2 Computer Science Department, University of Central Arkansas, Conway, Arkansas, USA; 3 Computer Science Department, University of Arkansas at Little Rock, Little Rock, Arkansas, USA; 4 The Georgia Neurosurgical Institute, Macon, Georgia, USA

## Abstract

**Background:**

Until quite recently spinal disorder problems in the U.S. have been operated by fusing cervical vertebrae instead of replacement of the cervical disc with an artificial disc.  Cervical disc replacement is a recently approved procedure in the U.S. It is one of the most challenging surgical procedures in the medical field due to the deficiencies in available diagnostic tools and insufficient number of surgical practices For physicians and surgical instrument developers, it is critical to understand how to successfully deploy the new artificial disc replacement systems. Without proper understanding and practice of the deployment procedure, it is possible to injure the vertebral body. Mixed reality (MR) and virtual reality (VR) surgical simulators are becoming an indispensable part of physicians’ training, since they offer a risk free training environment. In this study, MR simulation framework and intricacies involved in the development of a MR simulator for the rasping procedure in artificial cervical disc replacement (ACDR) surgery are investigated. The major components that make up the MR surgical simulator with motion tracking system are addressed.

**Findings:**

A mixed reality surgical simulator that targets rasping procedure in the artificial cervical disc replacement surgery with a VICON motion tracking system was developed. There were several challenges in the development of MR surgical simulator. First, the assembly of different hardware components for surgical simulation development that involves knowledge and application of interdisciplinary fields such as signal processing, computer vision and graphics, along with the design and placements of sensors etc . Second challenge was the creation of a physically correct model of the rasping procedure in order to attain critical forces. This challenge was handled with finite element modeling. The third challenge was minimization of error in mapping movements of an actor in real model to a virtual model in a process called registration. This issue was overcome by a two-way (virtual object to real domain and real domain to virtual object) semi-automatic registration method.

**Conclusions:**

The applicability of the VICON MR setting for the ACDR surgical simulator is demonstrated. The main stream problems encountered in MR surgical simulator development are addressed. First, an effective environment for MR surgical development is constructed. Second, the strain and the stress intensities and critical forces are simulated under the various rasp instrument loadings with impacts that are applied on intervertebral surfaces of the anterior vertebrae throughout the rasping procedure. Third, two approaches are introduced to solve the registration problem in MR setting. Results show that our system creates an effective environment for surgical simulation development and solves tedious and time-consuming registration problems caused by misalignments. Further, the MR ACDR surgery simulator was tested by 5 different physicians who found that the MR simulator is effective enough to teach the anatomical details of cervical discs and to grasp the basics of the ACDR surgery and rasping procedure

## Background

**Figure 1:**
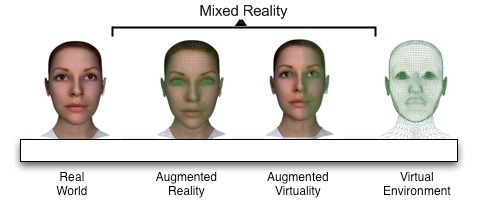
Milgram et. al [1] defines the distinctive worlds on virtual reality continuum

Mixed Reality (MR) provides users with an environment to perceive both the physical environment around them and the digital elements (virtual objects) presented through the displays (e.g. semitransparent displays). MR systems give users the illusion that digital objects coexist in the same space with physical objects.  For this delusion of coexistence, the virtual objects need to be precisely positioned and aligned with their overlaying real-world objects in real-time [1]. This alignment process is called registration. The registration of virtual and  real-world objects is a major feature of augmented reality systems and is the main challenge in such systems. Augmented reality (AR) is considered as a branch of MR which is a subclass of virtual reality (VR) [2]. According to Milgram et. al [2]  (see Figure 1),  an MR system includes both dominant physical reality and virtual reality aspects; however, an AR system includes more physical elements than virtual ones. According to Milgram’s definition the surgical simulation system presented in this paper is a MR simulation since our simulator has exactly the same number of real-world objects and their corresponding virtual objects. Interested readers are referred to a recent study of Costanza et. al [3] that surveys MR systems, applications, challenges and  current trends. MR has a highly interdisciplinary nature that engages fields like signal processing, computer vision and graphics, the design of displays, and sensors etc.

MR technology has been in existence for some time with applications in different areas such as manufacturing, architecture, military, visual media, and medicine [4]. A general technique to acquire motion tracking in these applications is using optical motion tracking systems such as VICON [5]. In a typical AR system, the user wears head mounted displays (HMD) which are the most widely used displays in MR [6]. The HMD forms a correct perspective view of virtual objects. Therefore, virtual objects are transformed and/or rotated according to the movements of the transformations and rotations of the HMD. This gives the user a sense of being present in the virtual scene. To transfer HMD’s transformations and rotations to virtual objects, tracking of HMD is necessary. In our simulator; however, tracking is done by stereoscopic binocular display and the user’s visual cue is only supported by what is shown on the binocular display. 

The outline of the paper is organized as follows: The following section presents applications of MR in medicine. This is followed by a background of artificial cervical disc replacement surgery (ACDR) and history of ACDR surgery in the US. Next, we introduce our MR simulation setting for ACDR surgery simulation and issues encountered in development of the simulator. Then we offer a solution to the main challenge in MR setting, the registration problem. Finally, we provide details on the experiments and the results obtained.

## Mixed reality in medicine

The deficiencies of current traditional education methods render virtual reality simulators as important instruments in medical training. The impact of traditional medical training is indicated in the report of National Library of Medicine (NLM) [7] with as many as 98,000 patient deaths in hospitals due to medical errors in the U.S.  Some of the errors are a result of surgical procedures and reveal insufficiency in medical training. Thus, the national priority in healthcare delivery is to reduce physician errors and as a result patients deaths. According to Merril [8][9], physicians experience higher error rates when they are newly practicing a surgical procedure. This fact is called a learning curve effect. In order to reduce this effect and let physicians gain necessary skills and experience, there is no current approach safer and more efficient than a virtual surgical simulator.

In the last two decades, MR has gained high recognition as an education tool complementary to the classical training methods in medicine and will possibly be a potential tool that increases the effectiveness of medical education [10]. Moreover, in the operation room, MR provides valuable insights by offering both a patient internal view (such as CT scan image projection for the particular area) and an external view that gives valuable and rich data to the surgeons to guide their actions [11][12-14]. Rosenthal et. al [15] showed that MR can actually improve a surgeon’s accuracy on needlebiopsy compared to traditional training methods.

A typical application for MR is microscope-assisted intervention MAGI [16] that presents 3D vessels obtained from pre-operative phase and superimposes 3D vessel image on microscope view. Hirsch [17] introduced another application that tries to overcome one of the main problems of interventional measures that arise from the limited visibility of the patient. Liu et. al [18] have summarized MR usage on particular operations such as orthopedic surgery, oral implantology, and laproscopic surgery. In another study, Welch et. al [19] introduced an MR system that uses multiple cameras to capture patient’s body and the captured body is then sent to a remote expert for patient screening.

## ACDR surgery 

More than 200,000 US citizens suffer from degenerative disc disease in cervical region (neck) [20]. Cervical disc replacement is one of the most challenging surgical procedures in the medical field due to the deficiencies in available diagnostic tools and insufficient number of verified surgical practices. In the U.S., spinal disorder problems have been operated by fusion of the cervical vertebrae for many years rather than the replacement of the cervical disc with an artificial disc. The first Federal Drug Agency (FDA) approved artificial disc replacement surgery for lumbar was made in June 2004. Quite recently (July 17th, 2007), FDA approved the first artificial cervical disc implant, the Prestige ST Cervical Disc System [21]. For physicians and surgical instrument developers, it is critical to understand how to successfully deploy the new artificial disc replacement systems. Without proper understanding of the deployment procedure it is possible to injure the vertebral body. Throughout the surgical procedure, activities such as compressions and decompressions on the vertebrae caused by new instrumentations that are specifically designed for the disc replacement operations need to be cautiously investigated. Also, stress and strain concentrations of life-critical contact locations on the vertebrae must be well comprehended. 

The ACDR surgery mainly consists of three phases; the removal of the disc material called a scooping procedure, adequately decompressing the nerve called a rasping procedure, and deployment of an artificial disc device into the prepared disc space respectively. The focus of this study is on the second phase; the rasping procedure. More specifically, the goal of this study was the creation of an accurate MR simulation of the rasping procedure in which the nerve is adequately decompressed and the vertebrae are raised up to an adequate height in order to embed artificial disc implant. After the rasping procedure the vertebrae surface and height become suitable for embedding disc implant. 

### Anatomical background

**Figure 2:**
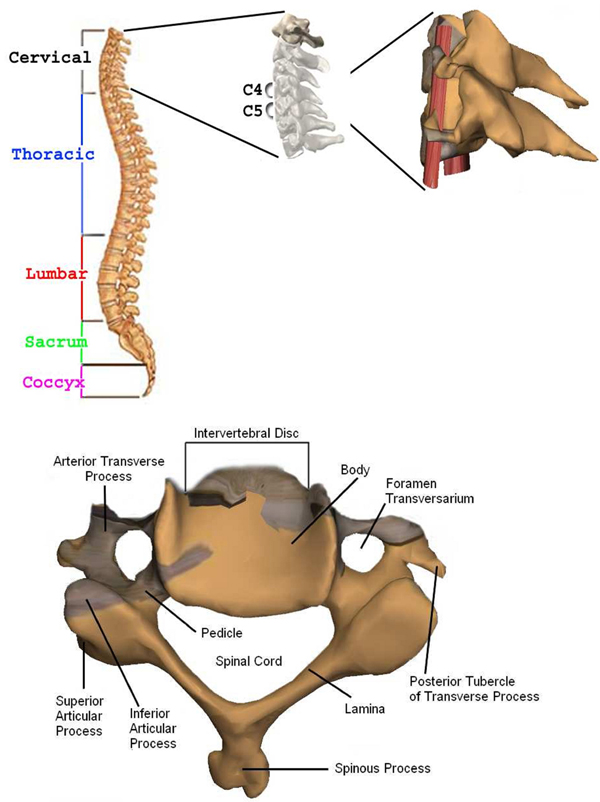
Top: Spine anatomy, cervical discs, real patient’s C4-C5 vertebra’s models (left to right), and Bottom: C4 vertebra

The section below the brain to the neck or to the thoracic spine is called Cervical Spine. This contains seven bones each abbreviated C1 to C7 vertebrae from top to bottom as given in Figure 2 [22]. The artificial disc replacement (ACDR) surgery deals with the cervical vertebra C3 through C7 that they lie in the neck [23-27].  On the left and right side of the each vertebra, there are small tunnels called Foramen Transversarium (see Figure 2). Through these holes (Foramina) two nerves leave the spine. The Intervertebral Disc resides directly in front of the Foramina. The center of intervertebral disc has a spongy material, called nucleus, that provides a shock absorption mechanism. The nucleus on top of the intervertebral disc is held by annulus which are like serration rings on finger print. A degenerate disc (bulged or herniated) narrows the Foramina and puts pressure on the nerve. This is called degenerative disc disease which is caused by ageing or stress and strain on the neck. With time the degenerate disc wears out and this causes the vertebra above the degenerated region to lose its original height towards the vertebra below. Because of the abnormal pressure on the joint, the articular cartilage, a slippery surface that covers every joint in the body ruptures. This is called arthiritis. The absence of articular cartilage results in generation of bone spurs. These spurs may fill up the Foramina that further narrow nerve openings. This causes pain and other symptoms in the arms and neck. Further the pain, weakness, and tingling in the arm can disable the patient. In some situations without surgery permanent damage may result. 

The ACDR surgery restores the normal distance between the two vertebrae so that it relieves pressure on the nerves and removes the symptoms. Unlike traditional anterior cervical discectomy and fusion methods for treating degenerative disc disease, the ACDR preserves natural motions of a healthy disc e.g. rotating and bending. Even though the ACDR surgery is extensively performed in Europe, only quite recently on July 17th, 2007 FDA approved the first artificial implant in the U.S., the Prestige ST Cervical Disc System [28]. The ACDR surgery is still a new procedure in the U.S. There are some possible risks associated with the ACDR treatment such as the bending or breaking of surgical instruments, wounds caused by applying extreme force on critical regions etc.  Any injury during the surgical procedure may lead to permanent damage.   The alleviation of all these possible risks by means of surgical simulation training is the main purpose of our framework. 

**Figure 3:**
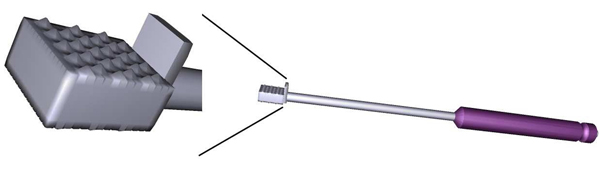
Our 3D model of the Rasp instrument and its tip in close view

**Figure 4:**
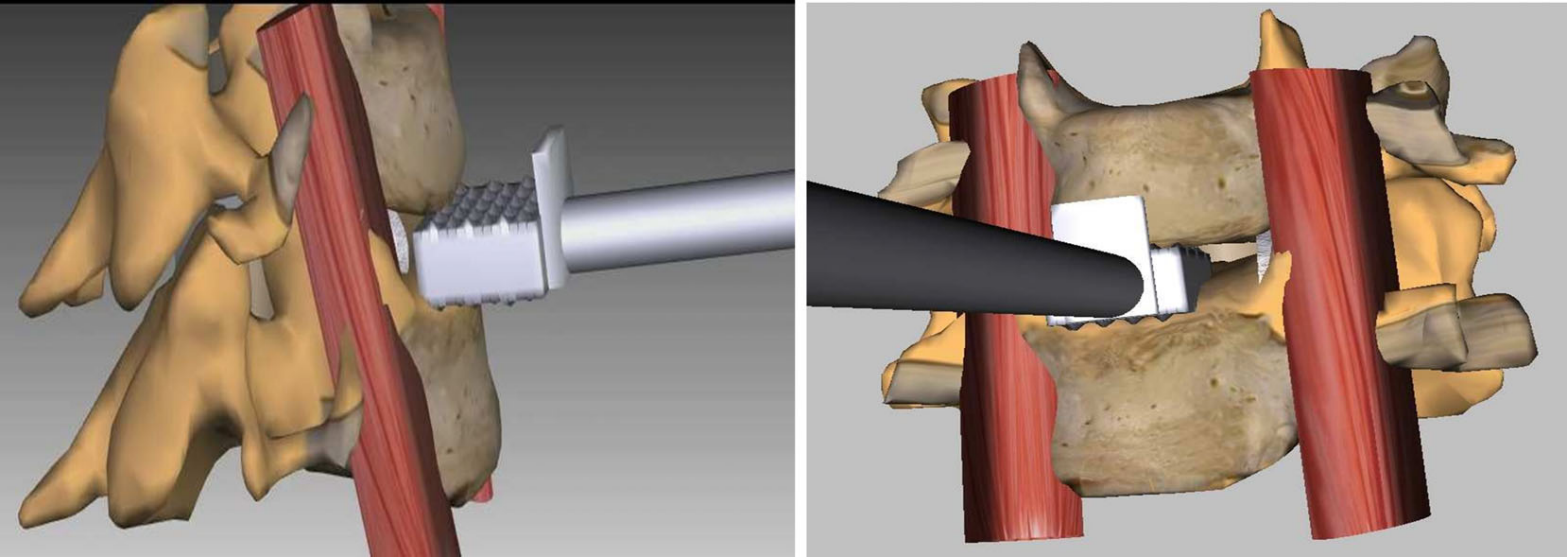
A procedure of intervertebral disc removal with the rasp in artificial disc replacement surgery

In ACDR surgery, rasping is one of the most important step. In this step the rasp instrument (see Figure 3) is used. Figure 4 illustrates exemplary rasping procedure between C4-C5 vertebrae.

For accurate MR rasping simulation, obtaining the critical forces applied on vertebrae by the rasp is vital.  Because the user is warned when he/she applies critical force, the  user learns proper application of critical forces.  In MR simulation this is achieved by finite element modeling of the rasping procedure.

## FEM analysis results and discussion

The finite element method (FEM) is a numerical analysis technique to obtain approximate solutions to engineering problems. Because of its accuracy and physical correctness as an analysis tool, it has received a lot of attention in medicine. For FEM analysis of the rasping procedure, specific attention is paid on the contact areas between nucleus and C4-C5. The real in vivo situation between the rasp and the nucleus has nonlinear contact nature. Therefore, the stiffness and damping parameters especially at contact regions are crucial for the realistic simulation.

Nonlinear contact element is used to simulate the real in vivo situation between the rasp and vertebra. The contact coefficient is adjusted with the comparison between the numerical simulation and experimental data. In order to implement a physically correct and plausible simulation, material properties (the Young’s modulus and Poisson’s Ratio) of the vertebrae C4-C5 were used  from Basa et. al [29]. 

**Figure 5:**
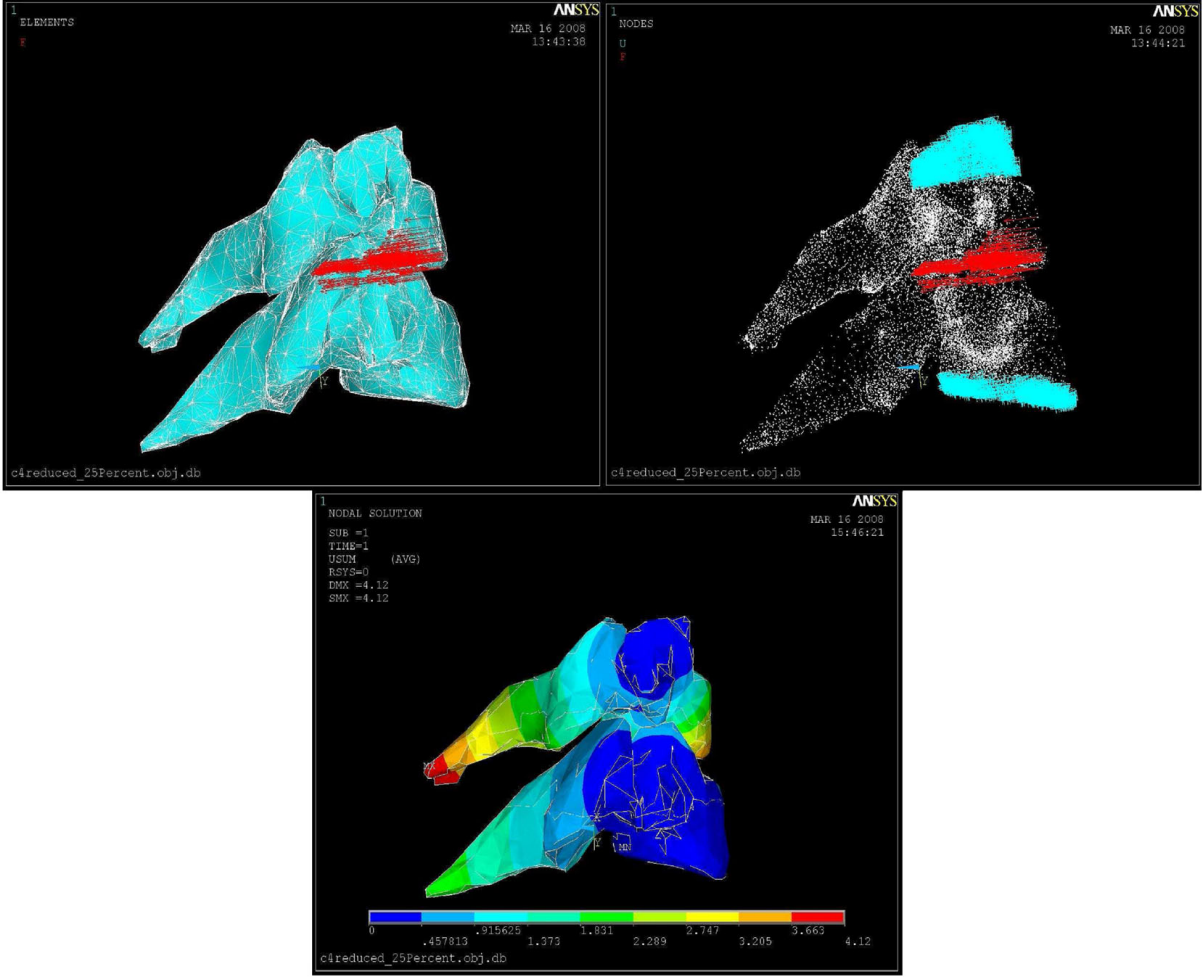
Boundary Conditions (Torques, left), Forces (Red, middle) on C4-C5, and displacement vectors (right): red indicates largest displacement

The FEM of cervical bones does not include disc since in an actual surgery prior to the rasping procedure disc nucleus and annulus are removed in the scooping procedure phase. Boundary conditions of C4 and C5 are determined based on the anatomical features given by a Neurosurgeon. As illustrated in the images (see figure 5), force vectors and boundary conditions are shown in red and torques colors in order. We had a total number of 16819 nodes, 80279 elements, 2544 constraint nodes, and 4061 contact nodes. The FEM modeling was done using the Ansys software.

**Figure 6:**
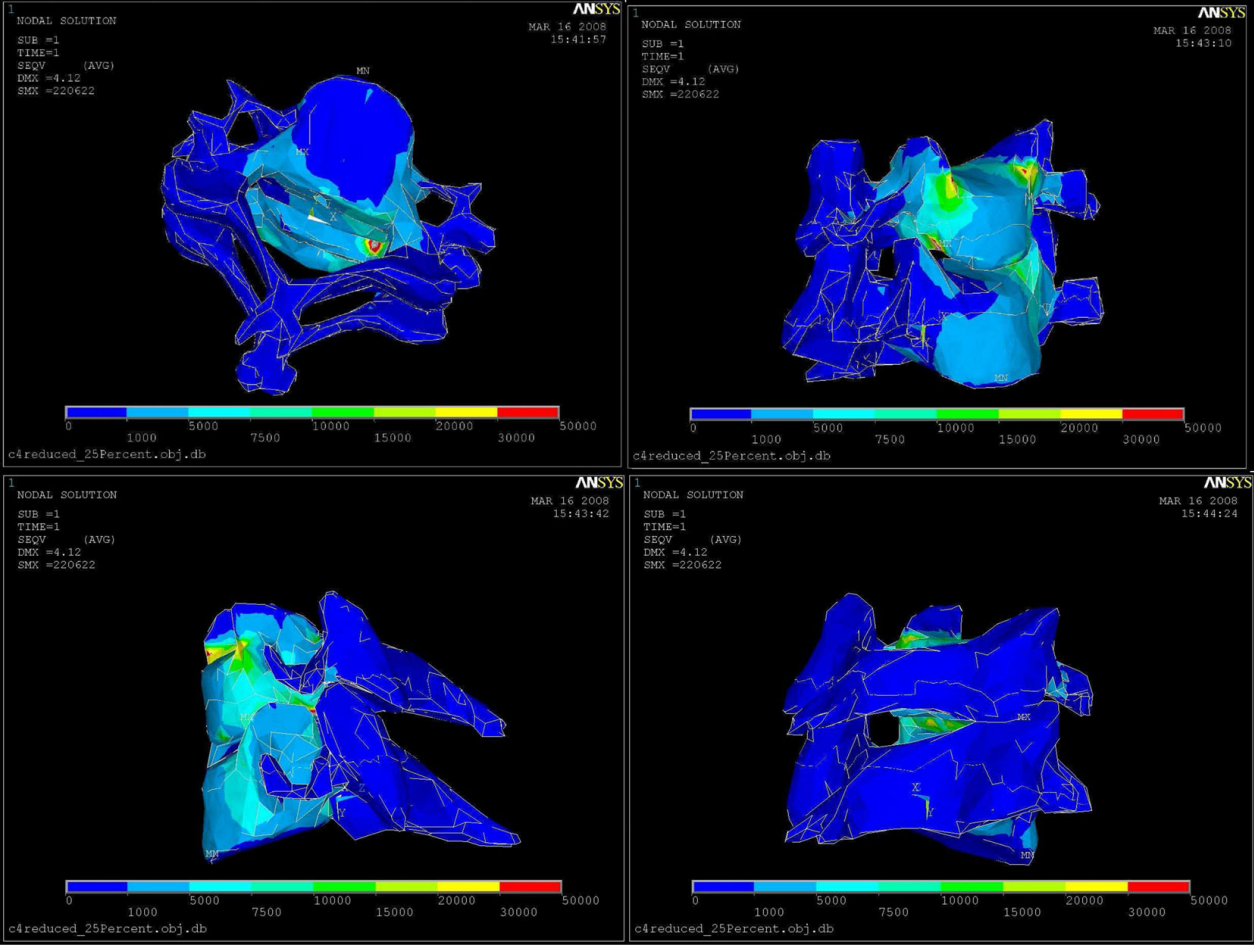
Von Misses Stress distributions: from blue to red stress is increasing.

After applying different forces, we found that 20 Newtons was the critical force to be applied throughout the rasping procedure. Larger forces than this critical force can harm the vertebral body endplate and the spinal cord. Any injury to the spinal cord can cause permanent damage. Figure 5 also illustrates displacement vectors which range from blue (smallest displacement) to red (largest displacement), boundary conditions (torques), and static force loading vectors (red). Von Misses Stress distributions for 20 Newton force loading is shown in Figure 6. Red color region on C5 in Figure 6 represents the critical area since this region is very close to the spinal cord.

## MR simulation setting

**Figure 7:**
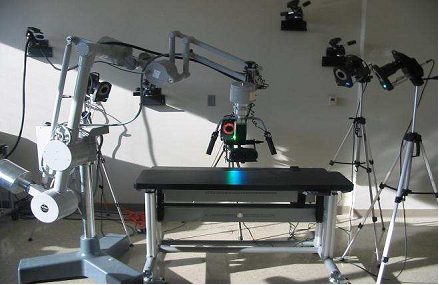
VICON Cameras on tripods and Microscope and binocular display and the end of the Microscope arm (Previous AR setting).

**Figure 8:**
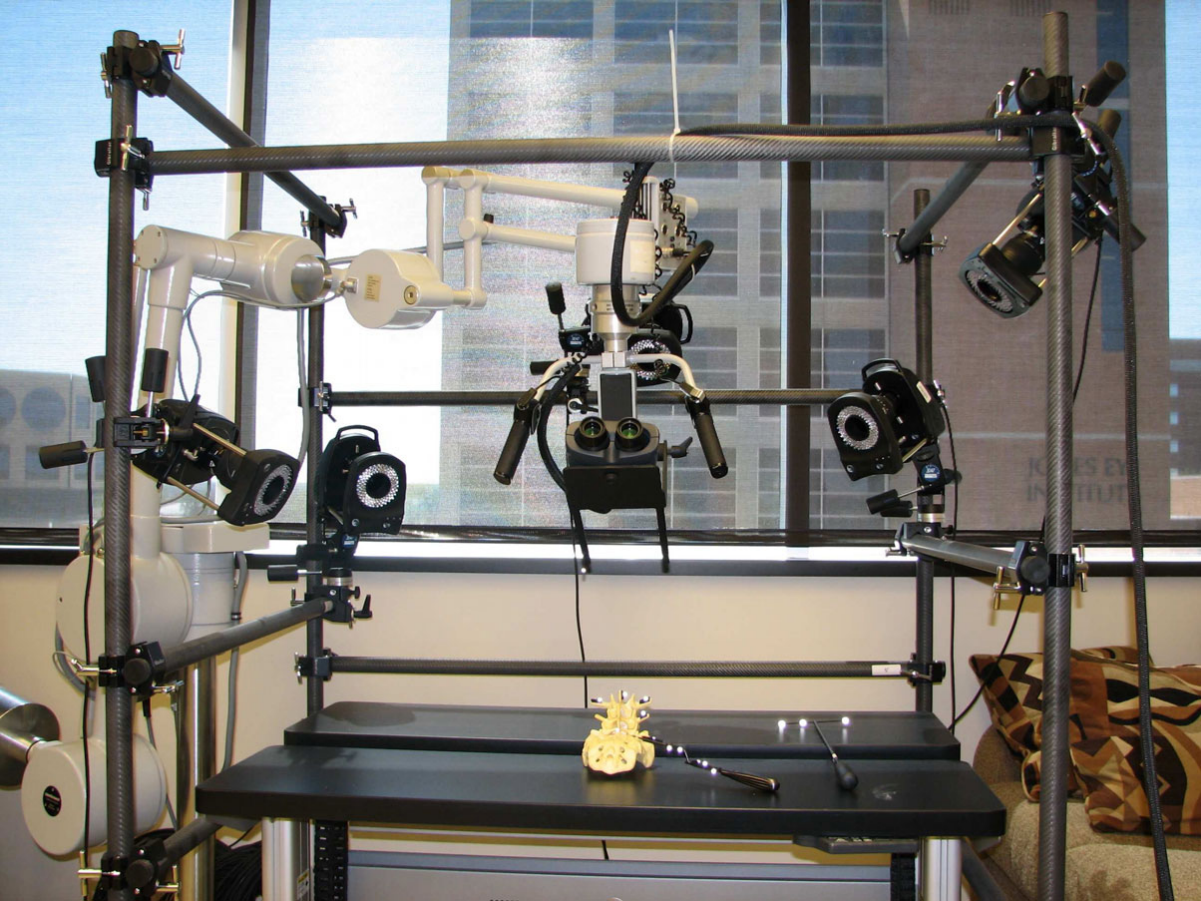
Current AR setting: Five VICON cameras on the bars, stereoscopic binocular display mounted binocular headgear. Plastic spine model and curette are placed within the tracking volume.

To create an MR system, motion capture and tracking are crucial steps where the user inputs and interactions are coupled. We developed our MR simulator using a VICON system to support “super-vision” and “super-sensing” for a user. The VICON is an active optical motion tracking system which allows us to track the motion of the surgical tools and plastic spine models in real-time. In principle, a tracking system consists of a set of devices which determine the position and orientation of real-world bodies (plastic mockup models and surgical instruments in our case). The set of devices in a tracking system acquires and interprets positions and orientations of these bodies. There are two types of tracking systems, passive or active. Active tracking systems are the most commonly used ones. In these systems, sensors are directly attached to the object to be tracked. The VICON is also an active tracking system in that objects are tracked via attached reflective markers. The goal is to accurately map the motions of the surgical instruments and plastic spine models to the graphics scene. Figures 7 and 8 show our previous and current MR settings respectively.

Our MR setting consists of 5 VICON cameras, a stereoscopic binocular display (NVIS Virtual Binocular SX^TM^), the robotic microscope handle for microsurgery to handle binocular display, real surgical instruments, a VICON Mx net console, two 16-CPU SGI Tezro visual workstation from where stereoscopic binocular display obtains graphics rendering, and a quad core PC workstation where VICON Mx net console is connected. Each VICON camera is connected to a PC workstation via VICON Mx net console. Both workstations are also connected to each other. The NVIS Virtual Binocular SX^TM^  is a handheld, adjustable, interactive, immersive display system with 24 bits 1280x1024 resolution microdisplays. In our setup, the Virtual Binocular SX is mounted to the robotic microscope arm (large white device in Figure 8) and tracked by VICON. 

Figure 7 shows our previous MR setting. In this setting cameras are held on tripods; however, because of the vibrations in the building, good calibration of the cameras could not be achieved. Calibration is a capturing session where the subject moves body (e.g. plastic model) with markers in front of VICON cameras so that the VICON system can predict the object's range of motion and calculate the lengths of the object. Thus, to overcome the building’s vibration problem seen in our previous setting, the VICON cameras were attached to a drum cage. As seen from Figure 8, VICON cameras are placed carefully on the cage to maximize over all viewing angle of all cameras so that each camera sees objects in the viewing volume. This in turn provides better calibrations and tracking capabilities. The cage also helped us to get approximately the same applied vibration s on each camera and thus the calibration quality was improved. The cage had a dimension of 190x121x152 cm and the capturing volume corresponded to a rectangular box of 76x35x63 cm in size.

Our current MR setting is illustrated in Figure 8. However, even with the current setting, there were minute vibrations that were causing calibration problems and reducing plausibility of the simulation. This problem was overcome by a simple low-pass filter. The VICON can capture the models defined with VICON IQ software. We attached a black metal binocular display holder to the robotic microscope arm .Two additional black hinges were mounted to the end of the binocular holder in order to create enough space to asymmetrically place markers that will represent binocular display’s virtual model in VICON. Hinges and holder were painted to black in order to reduce reflectivity because additional reflections create noise. Thus, whenever position and orientation of binocular display is changed, it will be tracked by VICON. We always placed markers on the subject asymmetrically as we saw that when markers are placed asymmetrically tracking performance increased. The asymmetric placement of markers helps VICON IQ to differentiate left from right

**Figure 9:**
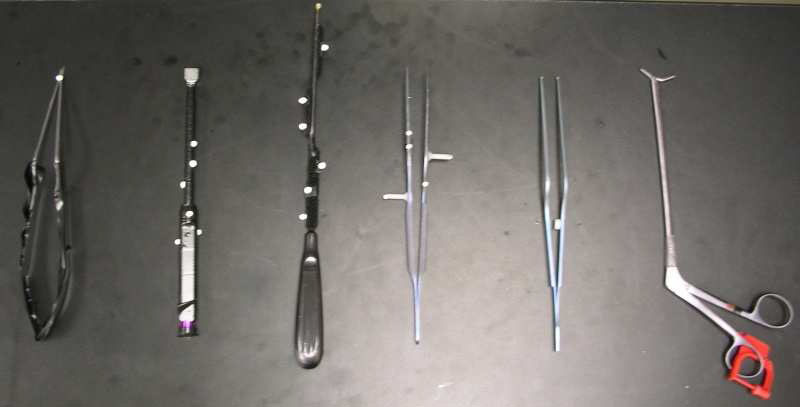
Common surgical tools: reflective markers attached to them for motion tracking purposes, the Rasp is second from left.

**Figure 10:**
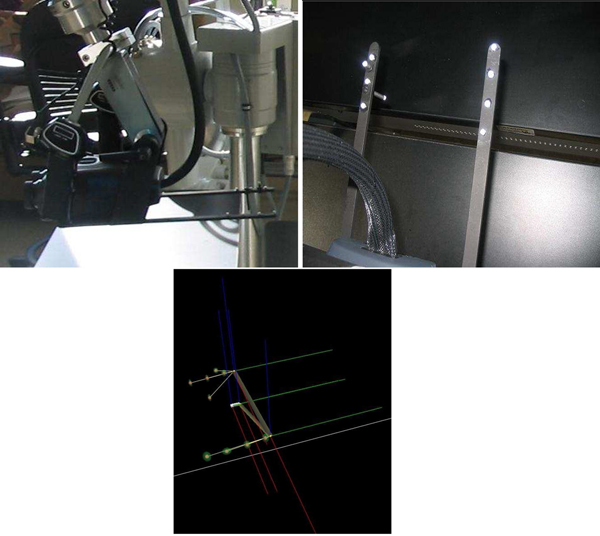
Left side view of the binocular display and right side hinges of the arm, and Binocular VICON Model (VST file).

Figure 9 shows surgical tools used in the ACDR surgery with attached reflective markers. In order to properly track small surgical instruments effectively, the markers reflectivity were surpassed by reducing reflectivity of the instruments with a black rubber band. Figure 10 illustrates physical environment and images of the stereoscopic NVIS Virtual Binocular SX^TM^ binocular display attached to microscope arms. 

The surgical instruments we used to track motions are particularly used for spine surgery. Optimum placement of markers on small size surgical instruments is a very challenging and tedious process since optimum positions for markers is found manually by trial and error. Whenever accurate motion tracking was obtained (optimum position is found) for a surgical instrument, markers were fixed in their positions. Otherwise, they were moved to another asymmetric position. Markers have a passive function with 0.3 to 0.5 mm in diameter and are made of a special retro reflective tape that echoes the incoming infrared lights. In Figure 10, right side shows the markers that spread across the hinges. Also, additional markers are located along the Z direction to differentiate the left arm from the right arm explicitly. Otherwise, the occlusion of markers along the left arm leads to an incorrect 3D construction of the model; in this case, it is likely to have a virtual binocular model upside down while the real binocular is in correct orientation. Figure 10 bottom image shows VICON model (VST file) for binocular headgear. This model represents real world binocular headgear geometry in a virtual domain. Figure 10 right side shows real-world binocular head gear and Figure 10 bottom image shows its corresponding virtual counterpart for tracking.

### System architecture and simulation loop

**Figure 11:**
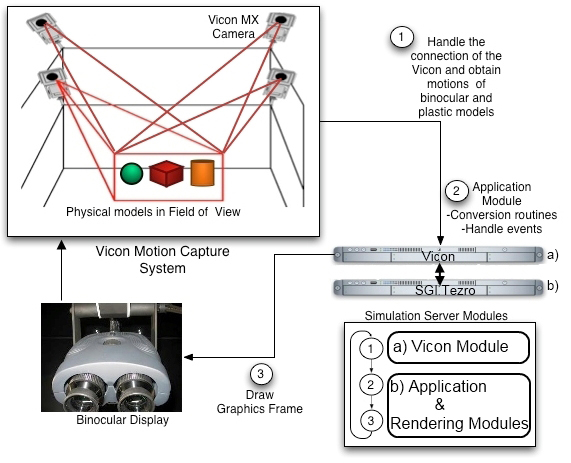
System Architecture and the Process Flow

In the developed MR simulator, the motion data is acquired from the VICON real-time engine before each of the rendering frame. Motions of surgical tools and plastic models are captured by VICON cameras and fetched by the VICON module via network. The VICON module basically establishes a connection between simulator server (SGI Tezro) and the VICON real-time engine called tarsus [30].  The position and rotations of the tracked objects are delivered to the graphics module inside SGI Tezro for graphics rendering in real-time. VICON computes the rotation of an object in exponential maps [30] which returns the degree of rotation as the magnitude of the vector. The vector itself signifies the vector of the rotation. The positions of the objects are delivered in millimetres that are converted in a proper scale for rendering. The connection through the VICON real-time engine, the proper scale conversion, and the rendering processes are managed by the VICON module, application module, and rendering module respectively.  Last two modules are in SGI Tezro. The schematic view of the system architecture and software modules is given in Figure 11. In the VICON tracking loop, the input stream is acquired in every frame. So, before drawing each frame, VICON client object requests the object positions from the VICON real-time server. Tracking frame rate is 200 Hz and the loop runs approximately in 30 Hz which satisfies our real-time performance requirement (30 frames per second). The gathered data is fed into corresponding VICON DCS (Dynamic Coordinate System). This coordinate system represents the coordinates in the capturing volume in the cage (field of view in Figure 11). VICON calculates the positions of the objects in millimetres and rotations in exponential maps. Translation data is scaled in centimetres and exponential maps are converted into quaternions.

For stereoscopic binocular display two different channels (channels as defined in OpenGL Performer) are created; one for the left eye and the other for the right eye. Each eye is managed by the corresponding video adapter. Also the right channel is attached to the left and distance offset is set to distance between two eyes. Since the surgeon focuses at hand depth throughout the real surgery, the focal depth of the binocular display is matched with approximate hand depth prior to beginning of the simulation.

Simulation is coded with SGI’s OpenGL performer graphics library. OpenGL performer is chosen because of its usefulness in stereoscopic rendering support by providing multi-pipe and multi-channel rendering. It is also optimized for performance that is a crucial part of all real-time simulations. All 3D models were constructed using the MAYA 3D modeling tool.

## Motion tracking with VICON

There are two different file types in VICON for modeling. One is VST that stands for VICON Subject Template, the other one is VSK that is for Calibrated VICON Subject. Actually, the common way to define an object is to design a general model for the object and create VST file. The next step is to calibrate the VST file according to the real-world object. However, this approach was developed for human motion tracking purposes. The human body parts have various degrees of freedom and actual marker position is dependent on other joints. For human motion tracking, it is not required to create VST because the markers should be in the positions where they are defined in modeling section. Thus, for human  motion tracking VSK files are created after a sample recording is captured. However, in our case, our VICON models (e.g VST files) were created fully manually. This was a meticulous and tedious process until the development of a semi-automatic registration scheme (see registration of models section for details). 

Although the binocular is a single rigid body, the model created for binocular consisted of several rigid bodies. According to the experiments conducted, building a model for each binocular handler hinge and connecting them with a rigid segment is more efficient than defining one whole rigid body. This also reduces flickering and jittering of the root segment when marker occlusion or overlapping occurs in the volume. With trial and error, the model was finalized as shown in Figure 10. Another issue is the covariance value that denotes likelihood of the marker to be located within the threshold. In the model, objects have joints and different degree of freedoms. Covariance is minimized because the binocular is a rigid body and all markers are supposed to be in the location where they are defined in model design (VICON model, VST file). Therefore, markers are not parameterized and are not dependent on joints. Also, all models are registered according to their root segments. 

For augmented reality purpose, a disposable C4-C5 model was used and their 3D geometries constructed from real CT scan images of a patient. So the metrics of the vertebrae were same as the real vertebrae itself. In addition to the vertebrae, all the surgical instruments’ 3D models were constructed from real instruments; hence, their metrics were exactly same as well. However, the rasp modeling was more challenging due to the width limitations on the surface. It is likely that markers on the rasp cannot be seen by any of the cameras that lead to unstable alignments of the real-world rasp to the virtual rasp. In this case, flickering and sudden turning upside-down orientation changes occur. The solution to that was to place markers across the side of the rasp that minimizes overall occlusion. Since throughout the ACDR surgery C4-C5 are almost free of movement, to give that sensation to the physicians our plastic C4-C5 model was molded in an ellipsoid box (see experimental results and discussion section for illustration).  The mold in turn provides object fixation and tracking vertebrae with the markers on the ellipsoid box. 

## Registration of models

Optical motion tracking for relatively large objects such as human body in a typical MR setting is easier than working with relatively small objects, since markers and real domain objects are big and useful for arbitrary placement of markers for human body tracking. However, in our case, considerable time was invested to accurately place the markers for achieving a close-enough reality. While this tedious placement process creates common basis up to a certain level with at least three markers’ placement (theoretically), the approach is still limited to an arbitrary placement scheme. Therefore, there is a necessity for more markers to be placed in order to increase accuracy and realism. Otherwise, lack of information about markers’ positions causes inefficient tracking and results in unrealistic simulations such as objects floating through each other, sudden change in orientation or jittering and flickering on rendered image of virtual scene due to the misalignments between real-world object and corresponding virtual object. This causes a continuing more elaborate and time-consuming placement process. Therefore, we developed a two-way semi-automatic MR marker placement and mapping system for optical tracking systems in surgery operations. Two-way implies extracting markers’ positions from developed software and alignment of the markers positions in a virtual domain from optically tracked real domain object. Semi-automatic implies user interaction of markers’ placement in the virtual domain.

In our simulator, registration errors arise from the discrepancies between the motion tracking system and graphics rendering system. This causes the users to see the wrong region of the 3D geometry while physically interacting with another region. Thus, even slight registration errors give wrong tactile feedbacks to the user (User obtains tactile feedbacks directly from his/her interactions with the plastic models).

**Figure 12:**
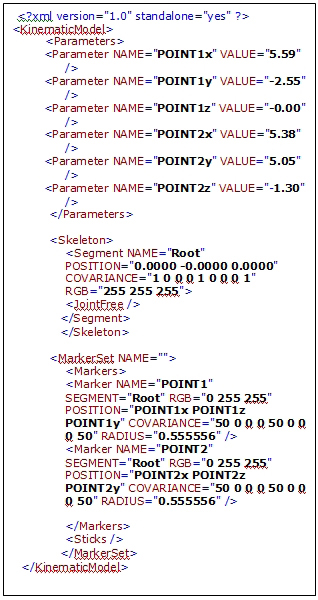
A sample VSK  file, generated XML file for VICON-IQ software

In the simulation, the physical objects (e.g.  plastic skull, C4-C5 vertebrae, surgical instruments; curette, rasp etc.) were tracked with VICON tracking system and visualized with our rendering module.  In order to track an object, VICON needs VSK extension files that articulate the features of the physical model.  Therefore, prior to the simulation, all the objects in the simulation should have corresponding VSK files. As described before, VSK file basically contains marker coordinates, joints and  segments in XML format (see Figure 12).

In order to automate the registration, our developed application generates the vsk file which makes the physical object ready for tracking via VICON. Figure 12 illustrates the output xml file created by our application. In the file, as mentioned earlier parameters include point names and their values correspond to coordinates. In the segment tag, we have one segment called root for this case. Also, this segment is a logical free joint and it represents the center of the object that has six degrees of freedom. Therefore, this root segment can freely move in the tracking volume. In fact, the root segment’s motion is captured with respect to the other points that are connected to itself. Marker Set tag enumerates all markers and joins them on the root segment. Radius tag denotes the marker’s radius which is 0.5 mm diameter. The generated file can be directly imported to VICON and then registration process will be completed. Since the markers positions are known, transformation is computed based on these positions. When the objects are tracked, transformation values are calculated relative to the given orientation. Thus, reflecting the object transformation to current virtual object will produce an aligned virtual and VICON model.

### Semi-automatic registration

We developed two different schemes for minimizing the registration errors. The first scheme is by placing the feature points directly on the virtual object and generating the VSK file within our application. The second method is to import the VICON file that contains marker positions to our application and then performing the registration by mapping the markers on virtual object. This second step   minimizes the error by calculating transformation and the rotation matrix based on least-squares method by single value decomposition scheme [13]. For details of least square approach for MR type of applications, reader is referred to our recent work [31]. One of the contributions of this study is to minimize errors in the registration with the algorithms and methods that best fit our case. Although our tool is highly complementary to VICON software, it could be used with any optical motion tracking software and tool as well.

Previously, the alignment of 3D computer models with tracking models was accomplished manually. With our approach and tool, the registration is performed at ease with the user interaction (semi-automatic). In order to track the objects, they ought to be defined and modelled prior to the beginning of the simulation with VICON’s modeling tool. The VICON system compels users to attach markers on physical objects and define their tracking model for the physical object. However, the whole task is entirely separated from the virtual object and that urged us to develop a solution so that the physical model can be associated with the corresponding virtual model and generate the tracking model file for the VICON system.

**Figure 13:**
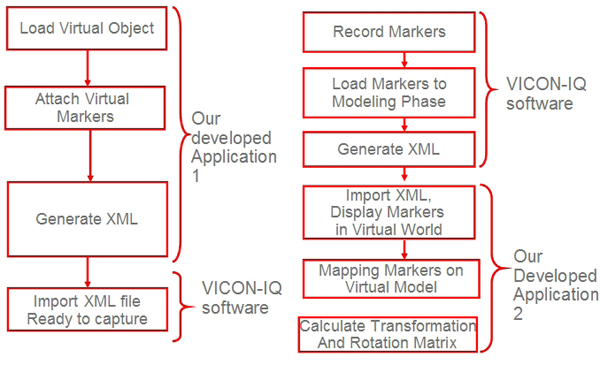
Left and right: 2 developed approaches for registration respectively

We developed two approaches for registration. In the first approach, we reversed the conventional way of creating tracking model file by determining the marker’s location on virtual object. The next step will be the attachment of physical markers on those locations. The first approach is illustrated in Figure 13 and details of this methodology are explained under the subject model file generation title. 

The second approach is a more general solution to the registration problem and can be easily applicable to other optical motion tracking systems. With this approach, markers locations are determined with a sample motion recording through VICON. The generated markers in xml file are imported to our tool. The mapping of these markers’ positions on virtual object completes the registration process. This final mapping of the markers requires a manual mapping by the user. Finally, registration offset and orientation divergence is calculated with the least square approach by our tool. Thus, registration discrepancy is decreased and the whole registration process can be quickly completed.  Moreover, re-registration of incorrect alignment can be carried out efficiently.

**Figure 14:**
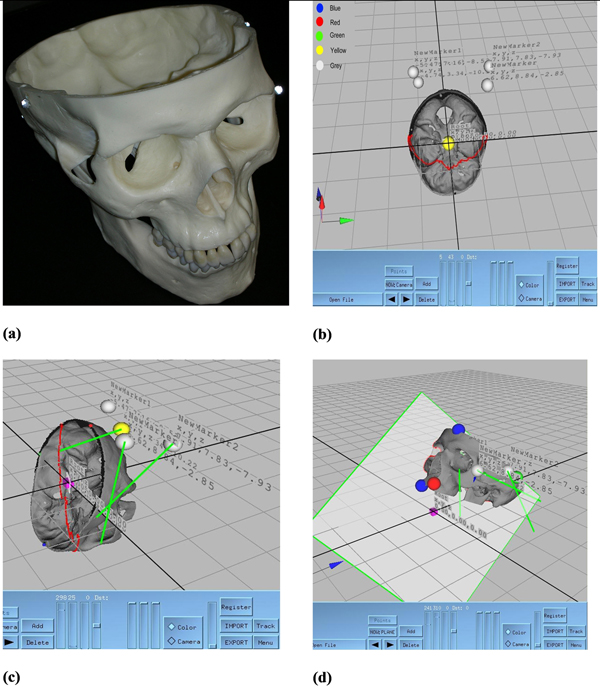
**Our semi-automatic registration scheme** (a) Physical object with reflective markers glued at its sides (b) Markers positions’ are imported to the developed software (c) Mapping of the markers accomplished (d) Registration is achieved, transparent surface framed in green colour is a reference plane. The red points are the intersection of reference plane with virtual object.

In Figure 14, the registration steps are illustrated.  In (a) the physical object with attached markers is captured. In (b), the file is imported to our tool. The grey spheres indicate the imported marker positions on real skull model. Then in our tool we manually associate each marker’s current position on the physical object to the virtual object (see (c)). The green lines represent this association. In (d) the virtual object is registered and VSK file automatically created.

The attached points’ positions are the locations of the markers on the plastic model. Figure 14 shows sample screen from our developed software which is directly connected to VICON IQ. Grey spheres are actual marker positions, yellow is currently selected marker, blue spheres are extreme points on cross section plane, and red and green spheres are extreme points of the skull object (The green, red and blue markers represent maximum and minimum vertices along x, y, z directions respectively). These points supports perception of the whole mode while locating the desired location and pinning with virtual markers. The purple cube shows the root segment or local origin of the 3D object. The root segment is placed on the origin initially and positions of markers are written out to XML file with respect to this location. The yellow spheres denote the markers placed by the user. The coordinates of each yellow marker are indicated on the screen with x, y, z triplets. These coordinates are given with respect to their local coordinate or root segment, which is (0, 0, 0) in this case. In order to help the user, in each marker assignment, the location is displayed on the screen. Finally, we can stick the markers at these locations on the real-world object for that object VSK file is already generated. 

Although, a minimum three markers should be positioned for tracking, for better precision, it is necessary to use more markers. However, when the area of body is small, putting more markers, especially too close to each other reduces the accuracy of tracking. With the help of the developed registration tool the tedious and time consuming registration process became a lot faster, easier, and more accurate. Previously, obtaining correct and accurate registration for the skull model took tens of hours; however, with the tool it took less than a minute with more accuracy. Thus, problems (flickering, jittering, sudden orientation changes) caused by misalignments are minimized. 

## Experimental results and discussion

**Figure 15:**
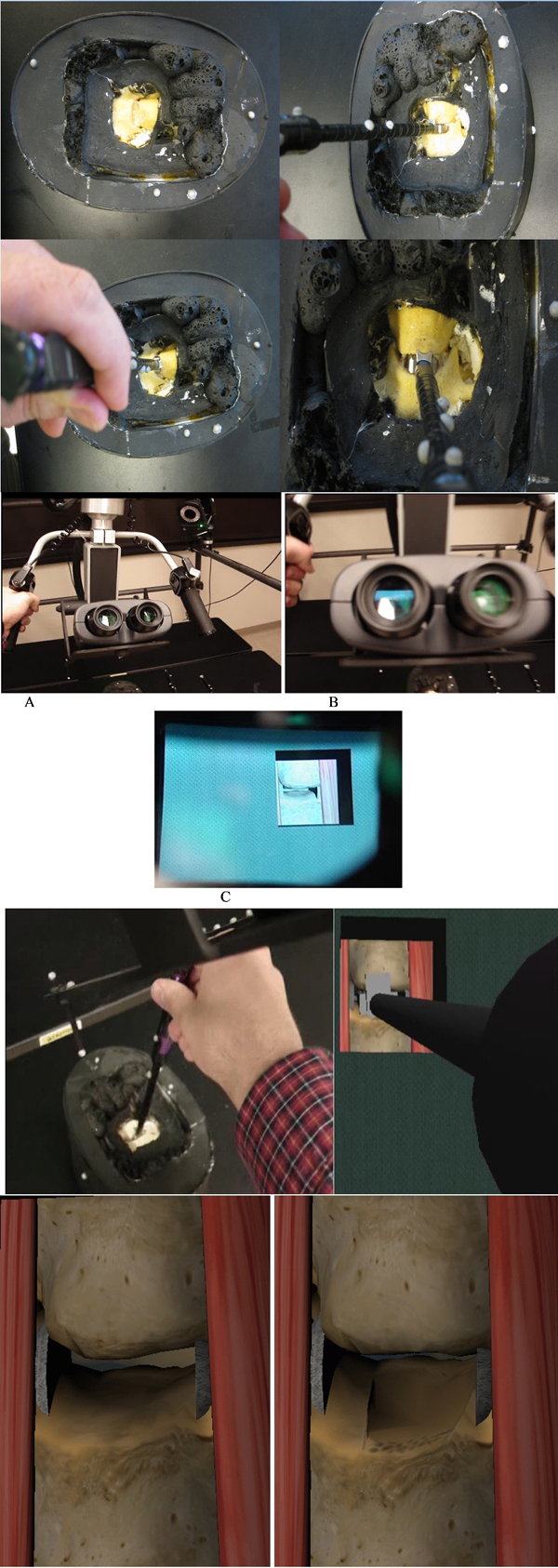
**Simulation screen: physical and virtual actors in the rasping simulation** (a) C4-C5 vertabrae plastic models in mold and physicians physical interaction by using the rasp, (b) A. Binocular display controlled by the user, B. Binocular stereoscopic view, C. What is seen from the binocular display, (c) Left: Physician interacts with C4-C5 vertebrae by using the rasp, right: what is seen from the binocular display, and (d) C4-C5 before and after rasping respectively.

Our prototype MR simulation system use real-time video capture combined with optical motion tracking. The software runs on SGI Tezro workstations. As illustrated in Figure 15 throughout the MR simulation C4-C5 plastic models were fixed in a mold to let them being tracked by VICON. The rasp instrument and the binocular microscope handler are other physical objects tracked by the VICON. As shown in Figure 15 we have their corresponding virtual models in the 3D scene. Figure 15 shows physical interaction of physician with C4-C5 spine model by using the rasp instrument. Physician’s interactions with C4-C5 by using the rasp are tracked and aligned with the corresponding virtual models. This is illustrated in Figure 15. Before and after the rasping procedure what has been seen from the binocular display is shown in Figure 15. 

Testing the effectiveness of our MR-based surgical training system seems like a natural step in evaluating the benefits of the system. However, this part is the most challenging part of the simulation. For MR system evaluators, it is unclear to tell how effective the MR simulator is at conveying the necessary information to the trainee. The proper measures for effectiveness of a MR system are diverse due to the different experiences of physicians.

Currently, we have to solely rely on the experienced physicians’ observations and perceptions about the simulator.  Thus, 5 physicians tested our simulator and found the MR simulation effective enough to teach anatomical details of cervical discs and to grasp the basics of the ACDR surgery and the rasping procedure. 

More specifically, foreseen benefits observed by 5 experienced physicians for MR ACDR simulator are summarized as following. With the current status, the MR ACDR simulator is capable of:

• Easing trainees’ transition to actual patients.

• Avoiding adverse events such as harming the vertebral body endplate and the spinal cord by providing applied force feedback (simulator displays warning message if applied force is greater than the critical force).

• Improving procedural success of physician by providing chance for repetitive experiments. This effect is known as volume-outcome relationship [34][35].

• Presenting uncommon and critical scenarios possible in the ACDR surgery.

• Allowing errors to trainees and let them reach their own conclusions which are not possible in traditional training.

• Exposing limitations to trainees in using actual ACDR surgery medical equipment.

• Improving trainees’ accuracy and timeliness to respond.

• Being used in planning or preparation of diagnostics for specific patients. For instance, planning the ACDR surgery for a specific patient by generating 3-D plastic models based on the anatomical data from the actual patient.

• Producing standardized testing to evaluate a trainee’s proficiency on the rasping procedure in the ACDR surgery.

In the future, quantitative measurements about effectiveness of the system may be possible with statistical analysis of tens of physicians with equally-good experiences (subjectively). From these experiments, it may be possible to perform statistical analysis and in turn occur at an overall performance measurement for effectiveness of our MR simulator. 

## Conclusion

The usability of VICON motion tracking system for MR surgical simulation development has been proved (see figure 15). A number of challenges encountered throughout the development and setup phases was solved. The camera's vibration challenge was overcome by mounting cameras on the cage setting and implementing low pass filters which remove noise in motion data but lead to an unimportant latency of motion throughout the visualization. Physically accurate modeling of the rasping procedure was achieved by doing finite element analysis. Critical forces caused by static force loadings of the rasp instrument were calculated. The object registration of the system has succeeded; however, the marker occlusion problem still persists. The findings from this study are expected to help train surgeons for the rasping procedure. This would also provide surgeons training environment for the rasping procedure and ultimately reduce human errors.

Although it has been shown that the VICON optical motion tracking system can be used in a MR surgical simulator for supporting actor of traditional surgical training, there are many tedious, time consuming and unpredicted challenges involved. Especially for microscopic scale surgeries such as brain surgery VICON MR setting alone is not an effective tool for training purposes. However, VICON MR setting can be used for anatomical studies and large scale surgery simulations such as orthopedic surgeries. It is belived by the authors that this work will provide the necessary basis and details for VICON based medical application developers. 

As a future direction, the entire ACDR surgery, – the scooping procedure, the rasping procedure, and implanting artificial disc into the vertebrae -, simulation is planned to be developed on our VICON MR setting. Even though there is approximately $250K capital costs associated with this surgical simulator development, this will possibly be amortized over a period of time while it is used for physicians’ training.

## Competing interests

The authors declare that they have no competing interests.

## Authors' contributions

SK and TH have made equal contributions to this study and designed and implemented all of the algorithms and hardware setup. All of the authors participated in the overall direction of the study. The original VICON based AR simulator idea came from RR, SK and TH wrote the manuscript.
